# Diagnosis and Genetic Counseling Before and After the Birth of Children With Joubert Syndrome and Beckwith-Wiedemann Syndrome

**DOI:** 10.7759/cureus.80677

**Published:** 2025-03-16

**Authors:** Yuri Hasegawa, Shoko Miura, Masayo Kagami, Sumito Dateki, Kiyonori Miura

**Affiliations:** 1 Department of Obstetrics and Gynecology, Nagasaki University Graduate School of Biomedical Sciences, Nagasaki, JPN; 2 Department of Molecular Endocrinology, National Research Institute for Child Health and Development, Tokyo, JPN; 3 Department of Pediatrics, Nagasaki University Graduate School of Biomedical Sciences, Nagasaki, JPN

**Keywords:** beckwith-wiedemann syndrome, chromosomal abnormalities, genetic diagnosis, joubert syndrome (js), prenatal diagnosis

## Abstract

The patient was a second child prenatally diagnosed with Joubert syndrome (JS) by ultrasound examination and family history of a first child with JS. After birth, the patient was also diagnosed with Beckwith-Wiedemann syndrome. Here, we report this case as a lesson on the importance of focusing on diagnosing the first hereditary disease and also considering the possibility of the development of a second genetic disease when providing treatment. We were able to confirm the diagnosis of both syndromes by detailed genetic testing after birth, allowing genetic counseling for future treatment and the next pregnancy.

## Introduction

Joubert syndrome (JS) is a syndrome characterized by multiple malformations, including absence or hypoplasia of the cerebellar vermis, expansion of the posterior cranial fossa, ventriculomegaly, polydactyly, occipital meningocele, and polycystic kidneys [1,2]. When diagnosed in the fetal period, ultrasound or fetal magnetic resonance imaging (MRI) will show a characteristic finding called the molar tooth sign (MTS), which reflects the absence or hypoplasia of the cerebellar vermis [3,4].

Beckwith-Wiedemann syndrome (BWS) is a congenital anomaly syndrome with three main symptoms: exomphalos, macroglossia, and gigantism. The classification of BWS mechanisms can be described as follows: (i) Epigenetic variants: gain of methylation at imprinting control region 1 or loss of methylation at imprinting control region 2 on the maternal chromosomes; (ii) Paternal uniparental disomy of 11p15.5; (iii) Cytogenetic abnormalities: translocation, inversion, or duplication; (iv) Variant of the maternal CDKN1C allele; (v) Unknown mechanisms [5]. Because the type and frequency of malignant tumors that occur in BWS vary depending on the genetic cause, it is advisable to continue searching for genetic causes. The most common ultrasound findings for patients with BWS include omphalocele, polyhydramnios, macrosomia (enlargement of the abdomen and head circumference), macroglossia, enlargement of internal organs, such as the liver and kidneys, macroplasia, and abnormalities of the kidneys and urinary tract [6-8].

Ultrasound findings showed multiple malformations in the patient, and because a previous child of the parents had been diagnosed with JS, the patient was diagnosed with the same condition. However, after postnatal genetic testing, the patient was diagnosed with BWS in addition to JS because he showed clinical symptoms (overgrowth and macroglossia) that were not specific to JS. This is the first report of a case of JS combined with BWS. Careful evaluation of the prenatal and postnatal findings allowed detailed genetic counseling.

This article was previously presented as a meeting abstract at the 2021 Annual Meeting of the Japan Society of Maternal and Fetal Medicine 27, 2020.

## Case presentation

A 36-year-old, gravida 2, and para 1 mother was spontaneously pregnant with her second child (the patient) and had been receiving prenatal check-ups at a clinic from the early stages of her pregnancy. At 24 weeks and four days of gestation, she was referred to our department for further examination because of polyhydramnios.

Genetic analysis and genetic counseling of the first child and their parents

Both parents of the patient had previously been diagnosed as heterozygous carriers of a TMEM67 variant. The first child was a boy and had been examined by a pediatrician at the age of three months with a main complaint of a mass on the back of his head. Computed tomography and MRI scans of his head revealed hypoplasia of the cerebellar vermis and MTS, and JS was suspected. We performed whole exome analysis using the now-seven-year-old first child’s peripheral blood DNA to determine the genetic variants. The identified variants were Variant1(V1):chr8:93780633-93780633;T>C (on GRCh38), exon8:c.755T>C:p.Met252Thr (TMEM67, KR709486.1) and Variant2(V2):chr8:93797457-93797457;T>C, exon20:c2087T>C:p.Leu696Pro. V1 was transmitted from the heterozygous father, and V2 was transmitted from the heterozygous mother. V1 was designated dbSNP/ClinVar: rs202149403 and classified as “Pathogenic/Likely pathogenic” in ClinVar [9]. There were no reports of V1 in jMorp [10], indicating that it is an extremely rare pathogenic genetic variant. V2 was not registered in the dbSNP/ClinVar database or jMorp. It is unknown whether the maternal V2 is pathological; however, considering the father's pathogenic variant, it was speculated that the characteristic physical findings of the child also reflected the maternal V2, so the child was considered to be compound heterozygous and was diagnosed with JS. We classified V1 and V2 as pathogenic variants according to the classification in the American College of Medical Genetics and Genomics (ACMG) guidelines [8]. V1 and V2 are classified as pathogenic very strong 1 (PVS1) and pathogenic strong 1 (PS1), respectively. Therefore, the variants were judged to be pathogenic, considering the rules for combining criteria to classify sequence variants with one PVS1 and one PS1.

Genetic counseling was provided regarding the results of the genetic testing of the first child, and it was explained that the probability of the second child developing the condition was 25%. At the time of genetic counseling, following the diagnosis of the first child with JS and the discovery that the parents were carriers, JS was not covered by preimplantation genetic testing for monogenic disorders.

Findings of fetal ultrasound and MRI examination

**Figure 1:**
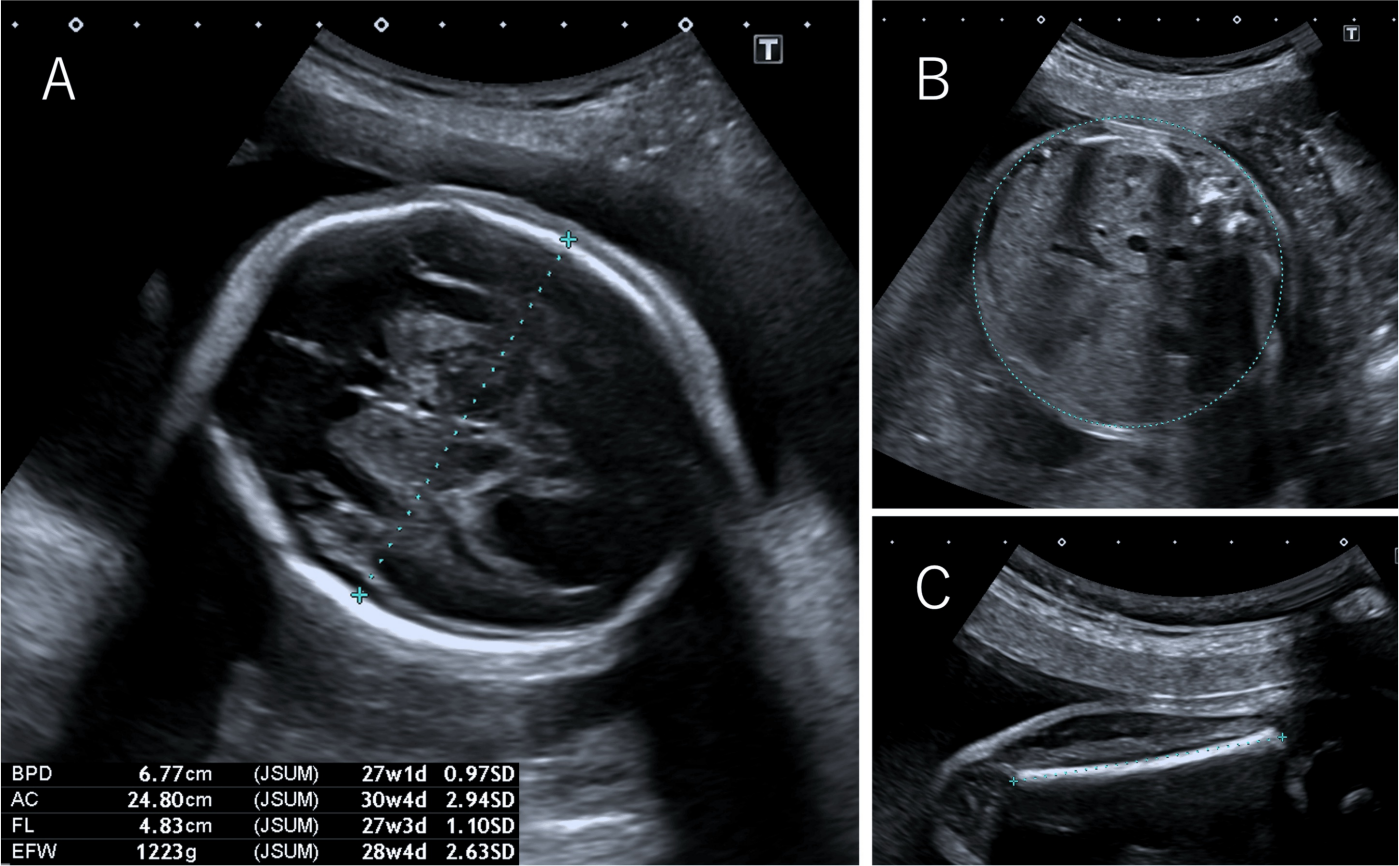
Ultrasound findings of the fetus at 24 weeks and four days of gestation. A. Biparietal diameter (BPD). B. Abdominal circumference (AC). C. Femur length (FL). The estimated fetal weight was 1.223 g (+2.63 standard deviation)

**Figure 2:**
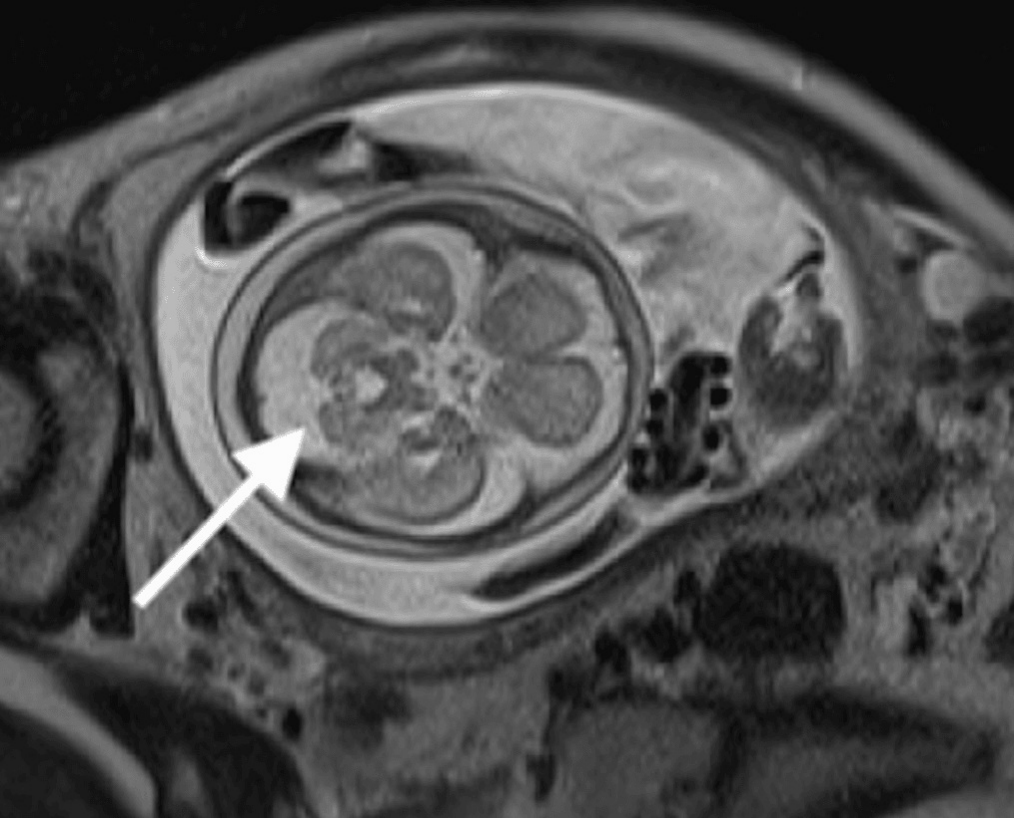
Fetal magnetic resonance imaging findings (T2-weighted image). The transverse image of the brain of the fetus shows the molar tooth sign (thickening of the upper cerebellar peduncle: white arrow).

**Figure 3:**
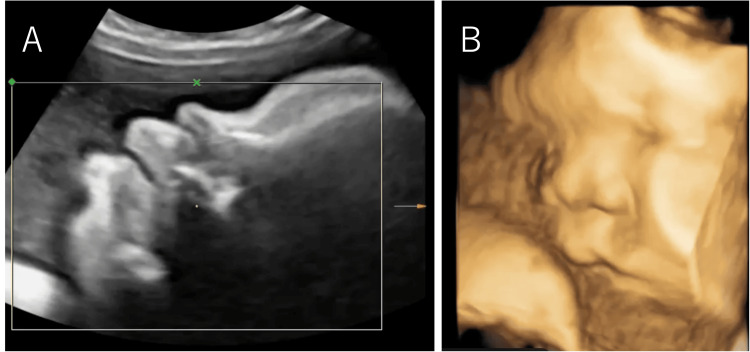
Ultrasound findings showing 2D and 3D images of the fetus’s face. A. Sagittal 2D ultrasound findings did not show any tongue protrusion. B. The 3D image also does not clearly show macroglossia.

Fetal ultrasound examination (Figure 1) showed macrosomia and a biparietal diameter = 67.7 mm (+0.97 standard deviation (SD)), abdominal circumference = 248.0 mm (+2.94 SD), femur length = 48.3 mm (+1.10 SD), and estimated fetal weight = 1.223 g (+2.63 SD). The amniotic fluid index was 27.9 cm, indicating polyhydramnios. In addition, the posterior cranial fossa was found to be enlarged at 12.4 mm, and the transverse diameter of the cerebellum was found to be small at 23.3 mm, suggesting cerebellar hypoplasia. The fetal MRI showed MTS (Figure 2, white arrow) and absence of the cerebellar vermis, which are characteristics of JS, as well as an enlarged liver. There were no apparent abnormalities in the digestive or urinary tracts. Ultrasound examination of the fetus's face showed no signs of tongue protrusion in either the 2D (Figure 3A) or 3D images (Figure 3B). Macrosomia, polyhydramnios, and liver enlargement are not typical findings for JS; we should have considered the complications of other diseases. The estimated fetal weight was +3.1 to +4.5 SD, and the amniotic fluid index was around 35. At 37 weeks of gestation, the baby was delivered by vacuum extraction.

Neonatal findings

**Figure 4:**
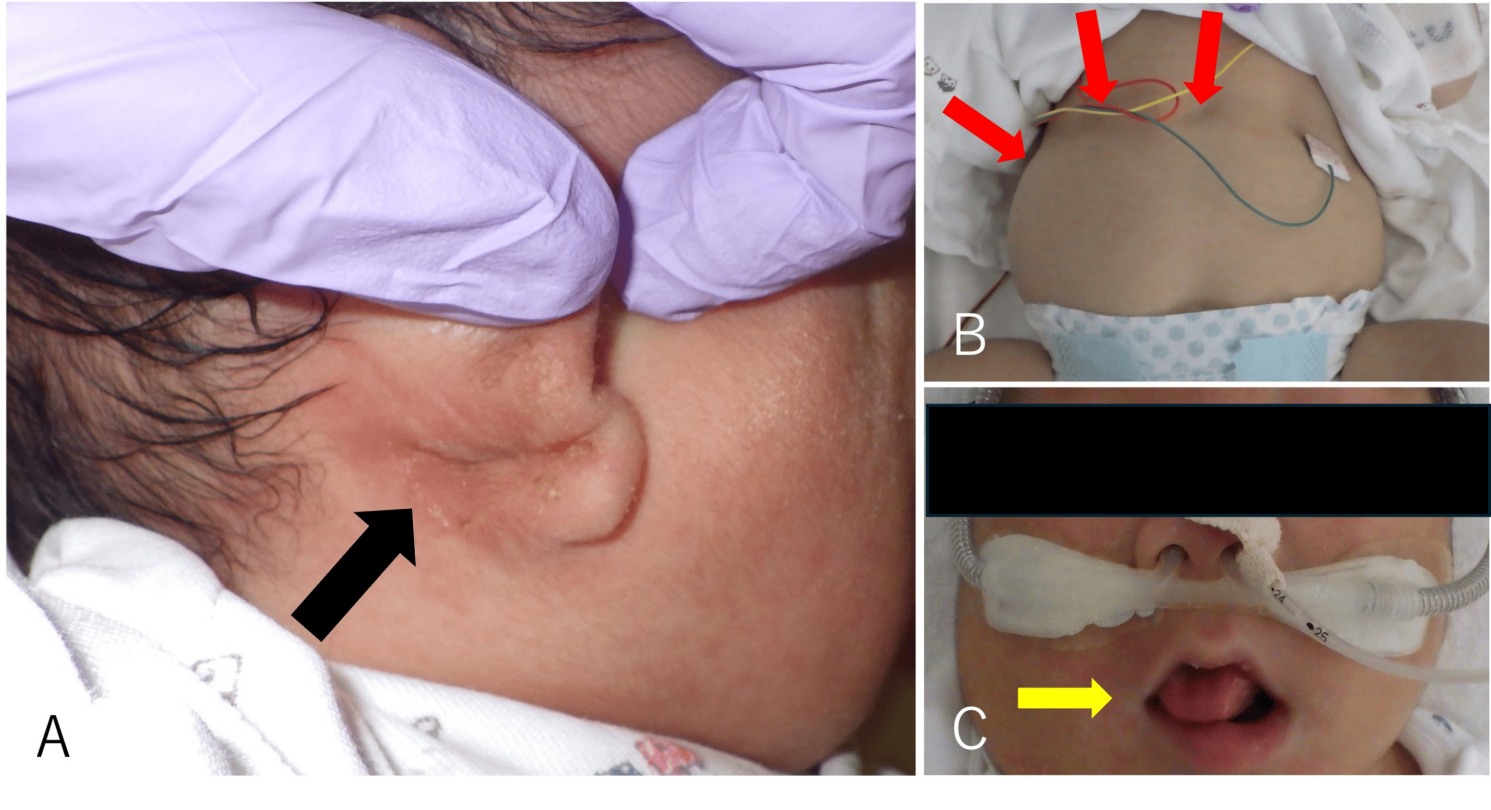
Neonatal findings. A. Ear creases and pits (black arrow). B. Abdominal distention due to hepatomegaly (red arrow). C. Macroglossia. The tongue protrudes from the lips (yellow arrow).

The baby was a boy weighing 4.326 g (+4.6 SD), with an Apgar score of 4/5 (1 min/5 min), umbilical artery blood gas pH of 7.25 and base excess of −2.5. The patient was born with weak crying and no muscle tone and was put on artificial ventilation. On examination, the patient was found to have ear creases and pits (Figure 4A, black arrow), abdominal distention due to hepatomegaly (Figure 4B, red arrow), and macroglossia (Figure 4C, yellow arrow). In addition to JS, the patient was suspected of having BWS, so genetic testing was carried out.

Genetic analysis of the second child

**Figure 5:**
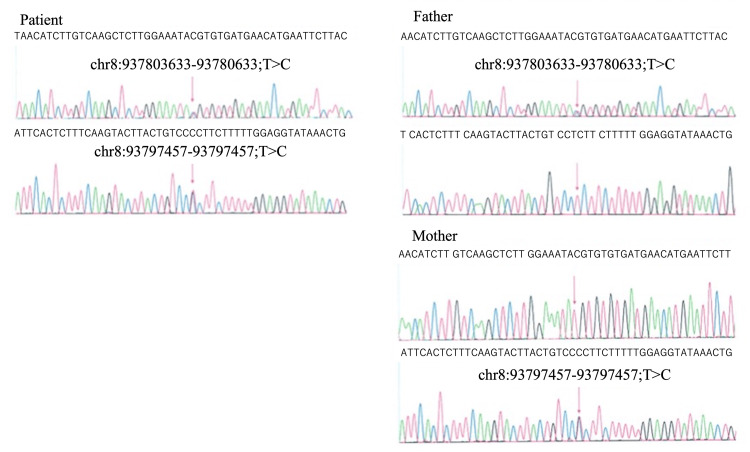
Single-exon analysis. The genetic mutations in the patient were found to be chr8:937803633-93780633;T>C, and chr8:93797457-93797457;T>C.

Single exon analysis of blood samples from the patient and parents was performed using a capillary sequencer. Two mutations, Mut1 and Mut2, were found in the patient, and he was diagnosed with JS (Figure 5). In addition, karyotype analysis of the patient showed 46,XY,der(11)(:p15.4→p15.5::p15.5→qter); the p15.4-p15.5 region of chromosome 11 was inverted and duplicated, and the p15.5-pter region was deleted. This result indicated a suspected duplication of the chromosomal region responsible for BWS. To determine the region of duplication or deletion and clarify whether it was inherited from the mother or father, array comparative genomic hybridization and methylation analysis were performed with the consent of the parents. Chromosomal examination was also performed on samples from the parents and their first child. Array comparative genomic hybridization showed partial duplication (4.27 Mb) in the 11p15.4-p15.5 region and partial deletion (457 kb) at the p.15.5 end. Methylation analysis showed hypermethylation of H19DMR and hypomethylation of Kv-DMR. The allele with the 11p15.4-p15.5 duplication was derived from the paternal allele. Finally, the patient was diagnosed with BWS. The karyotype of the parents and the patient's brother (first child) was normal.

Genetic counseling before and after the birth of the second child

**Figure 6:**
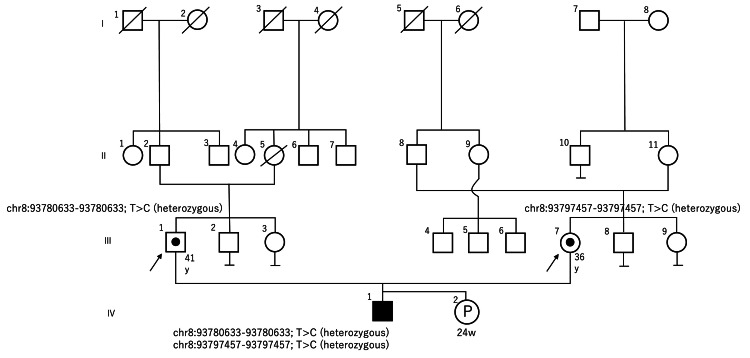
Family tree at the time of the first visit to our hospital. In this family tree, III-1 is the father and III-7 is the mother. There was no consanguineous marriage. The first child was diagnosed with Joubert syndrome through genetic testing, and the father and mother were diagnosed as carriers. Circle: female; square: male; filled square: male patient; crossed circle: deceased female; crossed square: deceased male; black circle in a square: male genetic carrier; black circle in a circle: female genetic carrier; P: pregnancy in this case; black arrow: clients.

The family tree at the time of the first visit to our hospital is shown in Figure 6. The mother conceived spontaneously, and when she visited the hospital, she was again explained the incidence of JS. At that time, the pregnant woman expressed her desire to raise the child, even if he or she had the same condition as the first child. At this point, general counseling for an autosomal recessive disease, JS, was provided.

After the birth of the patient, a detailed physical examination revealed an abnormality inconsistent with JS, and the results of detailed clinical examination and genetic testing revealed that BWS was present in addition to JS. It was thought that the BWS developed de novo, occurring at the same time as JS by chance and independent of JS. The mother and her husband had been diagnosed as carriers of JS, so she understood the diagnosis of JS in the patient. However, she seemed shocked to find out that her child also had BWS.

## Discussion

We report a case in which prenatal diagnosis of JS was performed on the basis of fetal ultrasound and MRI findings, but the findings were not specific to JS, and other diseases were suspected. The patient was diagnosed with a complication of both JS and BWS after birth. In this case, diagnosis of JS was based on the fact that the parents were heterozygous carriers of the TMEM67 variant and that a previous child had JS, as well as the fetal imaging findings. However, because macrosomia, polyhydramnios, and hepatomegaly are not characteristic findings of JS, and gestational diabetes had been ruled out, it was possible that other complicated conditions were present. The macroglossia characteristic of BWS was not apparent on ultrasound analysis, so BWS was initially not anticipated. In retrospect, in addition to BWS, conditions such as Perlman syndrome [11] and Simpson-Golabi-Behmel syndrome [10] should have been considered as potential diagnoses. Perlman syndrome is an autosomal dominant genetic disorder that presents with renal hamartoma in addition to macrosomia and hepatomegaly [11]. In this case, there were no abnormalities in the urinary system, including the kidneys. Simpson-Golabi-Behmel syndrome is characterized by overgrowth, a distinctive facial appearance, and the presence of multiple malformations from before birth [12]. The morphological abnormalities in Simpson-Golabi-Behmel syndrome overlap with the phenotype of BWS, such as macroglossia and umbilical hernia. Although the possibility of the simultaneous onset of other diseases with JS was present, comprehensive genome analysis would have been difficult. In the current case, findings not typical of JS were found in the patient by ultrasound examination. It is impossible to diagnose all diseases before birth, and it is not always desirable to inform the parents. However, it is thought that giving them a hint about the possibility of a disease is important in preparing them to accept the child.

On August 28, 2024, the Japanese Society of Obstetrics and Gynecology published a report on their website about the results of case reviews for preimplantation genetic testing for monogenic disorders in 2023 [13]. In order for PGT-M to be approved, it is necessary to provide information with the understanding that each case will be examined, not by disease type. Because JS is now included in the list of target diseases for preimplantation genetic testing for monogenic disorders, it will be necessary to provide the parents in this case with information again through genetic counseling in the event of a future pregnancy. In the current case, because we had been unable to diagnose BWS prenatally in the patient, it took time for the mother to accept the presence of the disease after the child was born. Clinicians should not be fixated on one genetic disease. If atypical findings are observed, further examinations and counseling may need to be conducted carefully after birth. After birth, detailed genetic testing for JS and BWS can be performed, and detailed genetic counseling can be provided on the recurrence rates of each disease; we intend to treat the child in this case accordingly and provide the parents with further information for future pregnancies.

## Conclusions

This is a rare case of JS and BWS occurring simultaneously, and although JS had been diagnosed prenatally, diagnosis of BWS before birth was difficult. When a given condition is suspected, it is important that the possibility of concurrent conditions is not disregarded if findings deviating from symptoms typical of the primary condition are observed. In this case, detailed genetic analysis after birth made it possible to provide appropriate genetic counseling to the parents.
